# Time- and dose-dependent correlations between patient-controlled epidural analgesia and intrapartum maternal fever

**DOI:** 10.1186/s12871-021-01249-1

**Published:** 2021-01-29

**Authors:** Bai-song Zhao, Bing Li, Qing-ning Wang, Jun-xiang Jia, Xing-rong Song

**Affiliations:** 1 Department of Anesthesiology, Guangzhou Women and Children’s Medical Center, Guangzhou Medical University, No. 9 Jinsui Road, Tianhe District, Guangzhou, 510623 Guangdong China; 2 Department of Anesthesiology, Women and Children’s Hospital, School of Medicine, Xiamen University, Xiamen, 361000 Fujian China

**Keywords:** Patient-controlled epidural analgesia, Maternal fever, Epidural duration, Anesthetic dosage

## Abstract

**Background:**

To investigate the relationship between intrapartum maternal fever and the duration and dosage of patient-controlled epidural analgesia (PCEA).

**Methods:**

This observational study included 159 pregnant women who voluntarily accepted PCEA. During labor, patients with body temperature ≥ 38 °C were classified into the Fever group, (*n* = 42), and those with body temperature < 38 °C were classified into the No-fever group (*n* = 117). The outcome measures included the duration of PCEA, number of PCEA, and total PCEA amount. Body temperature and parturient variables, including interpartum fever status and the duration of any fever were monitored.

**Results:**

The total PCEA duration and total PCEA amount in the Fever group were significantly higher than the corresponding values in the No-fever group (both, *p < 0.05*). The duration of fever was weakly correlated with the duration of PCEA (*R*^*2*^ = 0.08) and the total PCEA amount (*R*^*2*^ = 0.05) (both, *p < 0.05*). The total and effective PCEA were higher in the Fever group than in the No-fever group (both, *p < 0.05*). The total PCEA duration and total PCEA amount were positively correlated with the incidence of fever (both, *p < 0.05*). The diagnostic cutoff value for fever was 383 min, with a sensitivity of 78.6% and specificity of 57.3%. The mean temperature-time curves showed that parturients who developed fever had a steeper rise in temperature.

**Conclusions:**

This study showed that there were weak time- and dose-dependent correlations between PCEA and maternal fever during delivery. A total PCEA duration exceeding 6.3 h was associated with an increase in the duration of maternal intrapartum fever.

## Background

Intrapartum maternal fever refers to an oral temperature ≥ 38 °C during childbirth [1, 2]. Various prospective and retrospective studies as well as randomized controlled trials have shown that non-infectious fever during childbirth is associated with patient-controlled epidural analgesia (PCEA); about 20% of parturients that receive PCEA develop epidural-related maternal fever (ERMF) [1–3]. Our previous study confirmed this through accurate and continuous monitoring of core temperature, which revealed a maternal fever rate of 26.4% in women who used PCEA.

The specific mechanism of ERMF is not clear. It has been suggested that local anesthetics trigger non-infectious inflammation via immune regulation and cell injury. A single-center observational cohort study posited that ERMF occurred due to impaired release of anti-pyrogenic interleukin-1 receptor antagonist (IL-1Ra). The researchers investigated the effects of bupivacaine on apoptosis, caspase-1 activity, intracellular IL-1Ra, and the plasma IL-1Ra/IL-1β ratio in mononuclear leucocytes from laboring women. The study found that bupivacaine decreased the activity of caspase-1 in the peripheral blood mononuclear cells of laboring women, which subsequently inhibited the release of intracellular IL-1Ra by circulating leukocytes and revealed that immunomodulation by bupivacaine during labor promoted ERMF [4]. In two different studies, Wang et al. found that although the average analgesic duration in the early PCEA group was significantly longer than that in the late PCEA group, the average body temperature and fever rate were almost the same [5, 6]. They speculated that the relationship between PCEA and ERMF could be attributed to the “triggering effect” of local anesthetics via non-infectious inflammation. Consequently, they concluded that this effect was not associated with the duration of PCEA or the dosage of local anesthetics, although further research is needed to confirm the speculation. However, the relationship of epidural analgesia and maternal fever was not a relevant comparison. Investigations are limited in scope due to the existing body temperature monitoring methods. In most current investigations, maternal temperature is measured at predefined time points according to cervical dilatation [7] or at regular intervals during labor [6, 8]. Therefore, it is impossible to generate a precise and continuous temperature-time curve. Continuous monitoring of maternal core body temperature has not yet revealed the factors associated with elevated maternal temperature during labor.

This study investigated the associations of PCEA duration, anesthetic dosage, and intrapartum maternal fever using a novel method for the continuous monitoring of maternal core body temperature and the recording of analgesic data during labor. We focused on the effect of analgesia duration on fever, with the goal of generating a mean temperature-time curve to guide the clinical management of ERMF.

## Methods

### Research population

This study was approved by the Ethics Committee of Guangzhou Women and Children’s Medical Center (Protocol Number: 201939000). Pregnant women who planned to give birth during the daytime working hours at our institution between September 30, 2019 and December 31, 2019 were recruited for this study. Written informed consent was obtained from all participants before the start of the trial.

The inclusion criteria were as follows: 1) parturient with full-term singleton pregnancy (> 37 weeks); 2) scheduled vaginal in-hospital delivery; 3) vertex presentation; 4) no risk of mother-to-child transmission of an infectious disease; 5) no suspected fetal coagulopathy; 6) willingness to use PCEA and ability to provide informed consent; and 7) pre-epidural temperature > 37 °C. The following exclusion criteria were applied: 1) need for vascular access in both upper extremities; 2) absent upper extremities; 3) history of myocardial infarction, heart failure, maternal hepatitis, or HIV infection; 4) history of serious skin allergies, allergy to silicone or plastic; 5) preeclampsia; 6) taking paracetamol within 6 h before the study; 7) presentation other than vertex (i.e., breech or transverse); 8) gestational age < 37 weeks; or 9) suspected fetal coagulopathy.

On admission to the delivery room, all eligible parturients were informed about the study by the researchers and consent was obtained for study participation. The individuals collecting the body temperature data were blinded to the duration and dosage of PCEA.

### Body temperature measurement and treatment of fever

The axillary temperature was recorded (10 measurements per second) throughout labor using a smartphone/iPad-connected wireless thermometer (iThermonitor; Rui Ren Medical, China). The iThermonitor was secured to the (shaved) axilla using a hypoallergenic adhesive patch. The women were instructed to adduct the ipsilateral arm for up to 5 min after the iThermonitor had been secured, or until the temperature displayed on the paired smartphone/iPad was stable. Subsequently, the women were allowed to move their arms freely [9]. Unwarmed intravenous (IV) infusions in the contralateral arm were assumed to have had no effect on the measurement of axillary temperature with the iThermonitor.

Midwives monitored the parturient and followed the doctors’ prescriptions. The onset of labor was self-reported by the mothers. Fever was defined as an axillary temperature ≥ 38 °C. If the parturient developed a fever, the following measures were taken to lower the body temperature: when the axillary temperature rose to 38–38.5 °C, fluid replacement was also accelerated to replenish the water lost at this temperature. Acetaminophen (Johnson & Johnson Pharmaceuticals, Ltd., China) was administered orally to the women when the axillary temperature rose above 38.5 °C.

### Patient-controlled epidural analgesia

Epidural analgesia was performed when the contractions became regular and cervical dilation was up to 1 cm. Women receiving PCEA were given IV Ringer’s lactate 2 ~ 4 mL/kg/h. Rescue atropine and ephedrine were available at the bedside. An epidural catheter was introduced at L3–L4 and the epidural analgesia was patient-controlled. After 5 min, a 10 mL bolus of ropivacaine 0.0625% (lot number: H20140763; Astra Zeneca, Sweden) plus 0.3 μg/mL sufentanil (batch number: H20054256; Yichang Renfu Pharmaceutical, China) was administered. Then, the epidural catheter was connected to a pulse analgesia pump with the following parameters: loading dose, 10 mL; injection rate, 6 mL/h; PCEA (demand) dose, 8 mL; maximum dose, 40 mL/h; and lockout interval, 10 min. The analgesic level was assessed according to the patient’s sensitivity to temperature (ice) after 15 min. Additional 5 mL boluses of 0.1% ropivacaine were given for breakthrough pain and a visual analogue scale pain score < 3 at any time during labor.

### Data collection

Maternal demographic and clinical characteristics were recorded, including age, height, weight, body mass index (BMI), gender, and ethnicity. Analgesic data, including the duration of PCEA, total and effective number of PCEA doses, and total PCEA dose were also collected. The body temperature and changes in the parturients, including intrapartum fever status and the duration of any fever, were recorded. The data for parturients who were converted to cesarean sections were not analyzed. Maternal blood pressure and heart rate were monitored continuously during childbirth and the rate and volume of fluid administered were measured.

### Statistical analysis

Our previous study (in the process of review) showed that the ratio of fever and no fever in parturients during labor was 1:3. Based on this finding and the following formula with α = 0.05 and a power of 0.9, the minimum sample size required for the No-fever group was 111 and that for the Fever group was 37.

$$ {\mathrm{n}}_{\mathrm{C}}=\frac{\left(\mathrm{r}+1\right){\left({z}_{1-\beta }+{z}_{1-\alpha}\right)}^2{\mathrm{S}}^2}{r{\left(\varDelta -\delta \right)}^2}=\frac{\left(3+1\right){\left(0.84+1.96\right)}^2\times 47277}{3{\left(20-136\right)}^2}=37 $$

Note: r is the ratio between the experimental group and the control group, *Δ* is the superiority limit, *δ* is the difference between the mean values of the two groups, and S is the combined variance of the two groups.

Statistical analysis performed using SPSS software (ver. 21.0; SPSS Inc., USA) showed that gestational age, BMI, total PCEA duration, total PCEA dose, total number of PCEA uses, and effective number of PCEA did not conform to normal distributions; therefore, these data were expressed as the median and interquartile range. The groups were compared using the rank sum test. The correlations of the duration of fever with the total duration of PCEA and the total PCEA dose were analyzed with the Spearman method, and the effects of different factors on intrapartum fever were determined using stepwise logistic regression. The association of PCEA time with the diagnosis of fever was assessed through receiver operating characteristic (ROC) curve analysis. *p < 0.05* was considered statistically significant.

## Results

### Study subjects

**Fig. 1:**
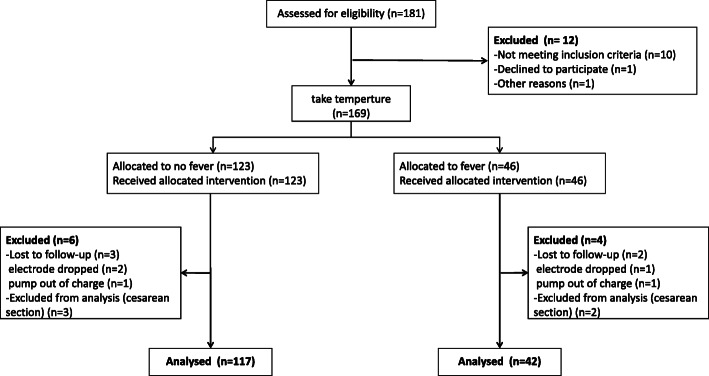
The flow chart for the study

This study included 169 women, among whom 46 developed intrapartum fever (Fever group, > 38 °C) and 123 did not (No-fever group, ≥38 °C) (Fig. 1). Finally, the data for 42 women in the Fever group and 117 women in the No-fever group were analyzed.

There were no significant differences in the maternal demographics (gestational age and BMI) or clinical characteristics (primipara/multigravida) between the two groups (Table 1). The total PCEA duration in the Fever group (9.70 ± 3.22 h) was significantly higher than that in the No-fever group (8.35 ± 3.98 h) (*p* = 0.049). The total PCEA amount in the Fever group (130.50 ± 73.22 mL) was also significantly higher than that in the No-fever group (99.87 ± 48.46 mL) (*p* = 0.003). However, there was no difference in the total PCEA amount/PCEA duration between the two groups (13.37 ± 4.62 mL/h vs. 12.84 ± 5.78 mL/h, *p* = 0.591, Table 1).

**Table 1:** Maternal and neonatal demographic and clinical characteristics

	Fever (*n* = 42)	No fever (*n* = 117)	Z/*t*/*x*^2^	*P*
Gestational age (week)	40.14 (1.50)	39.86 (1.50)	−1.182	0.237
BMI (kg/m^2^)	26.44 (2.90)	25.87 (3.60)	−1.592	0.111
Primipara/Multigravida	31/11	73/44	1.780	0.182
Total PCEA duration (h)	9.70 ± 3.22	8.35 ± 3.98	1.983	0.049
Total PCEA amount (mL)	130.50 ± 73.22	99.87 ± 48.46	3.041	0.003
Total PCEA amount /PCEA duration (mL/h)	13.37 ± 4.62	12.84 ± 5.78	0.538	0.591

Data are medians (interquartile range)

*BMI* body mass index

**Fig. 2:**
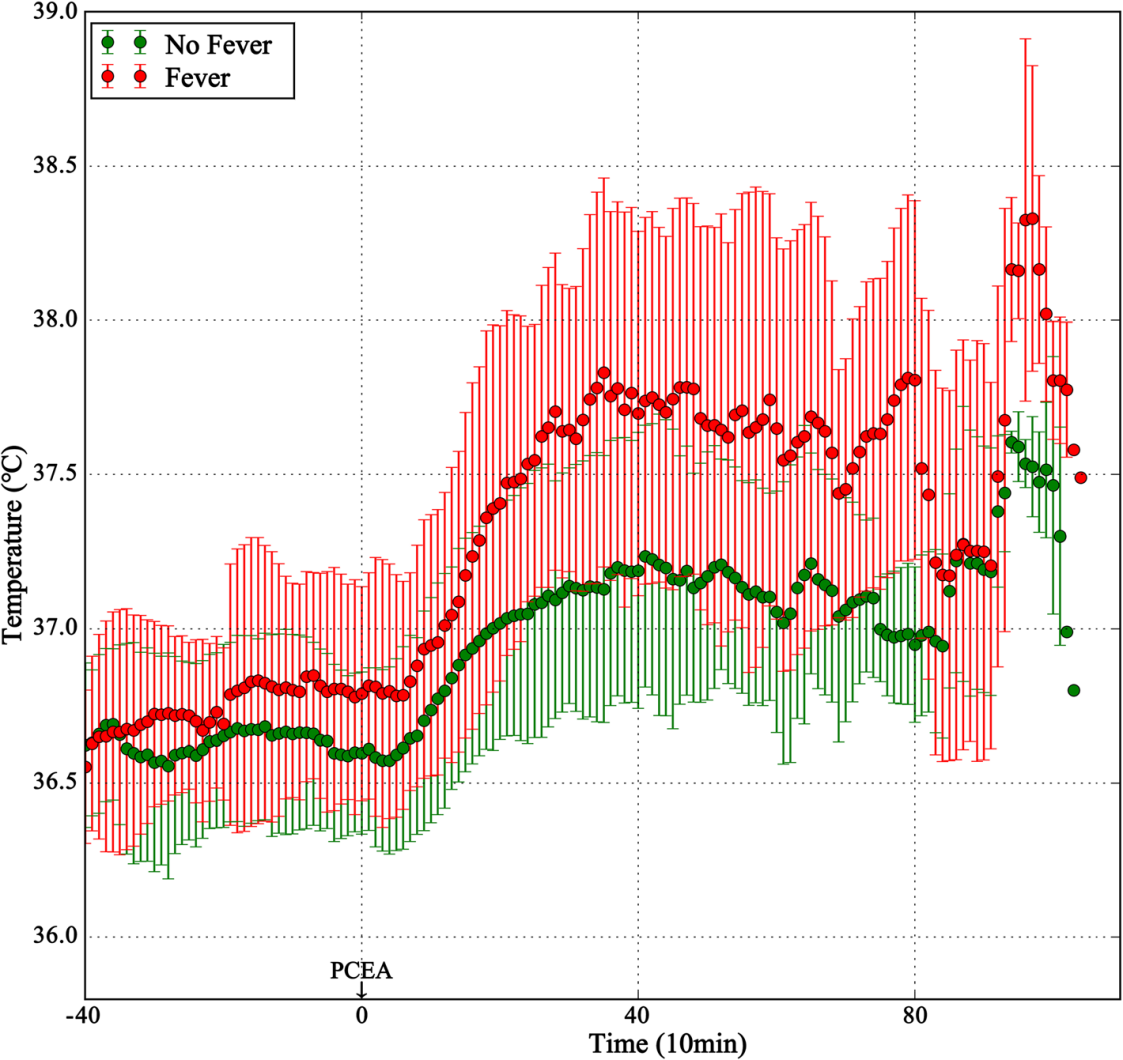
The mean temperature-time curve of parturients in two groups

The mean temperature-time curves for the Fever and No-fever groups showed that parturients who developed fever had a steeper, steady increase in temperature than those who did not develop fever, with a flat curve (Fig. 2).

Spearman correlation analysis showed that the duration of fever (1.53 h [1.16]) was positively correlated with the total PCEA duration (9.70 ± 3.22 h, *R*^*2*^ = 0.08) and the total PCEA dose (130.50 ± 73.22, *R*^*2*^ = 0.05) (both, *p < 0.05*), which indicated that the duration of fever increased slightly with the total PCEA duration and dose (Table 2).

**Table 2:** Correlations of duration of fever with total duration and dose of PCEA (*n* = 159)

		Total PCEA time	Total PCEA amount
Duration of fever	r	0.279	0.223
	*R*^*2*^	0.08	0.05
	*P*	< 0.001	0.005

In the Fever group, the parturients’ demand for analgesia was higher, and the total and effective number of PCEA were higher in the Fever group than in the No-fever group; the differences between the two groups were significant (all, *p < 0.05*) (Table 3).

**Table 3:** Total and effective number of PCEA uses

	Fever (*n* = 42)	No fever (*n* = 117)	Z	*P*
Total number of PCEA uses	3.00 (4.00)	2.00 (3.00)	−2.021	0.043
Effective number of PCEA uses	2.00 (2.00)	1.00 (2.00)	−2.290	0.022

Data are medians (interquartile range)

*PCEA* patient-controlled epidural analgesia

Stepwise logistic regression analysis was performed with intrapartum fever as the dependent variable. The total PCEA duration and amount had positive associations with the incidence of fever (both, *p < 0.05*). As the total PCEA duration and dose increased, the likelihood of fever increased (Table 4).

**Table 4:** Logistic regression analysis of factors associated with intrapartum fever (n = 159)

Risk factors	Regression coefficient	Standard error	Wald	*P*	OR	95% CI
Total PCEA duration (h)	0.002	0.001	6.181	0.013	1.002	1.000 ~ 1.004
Total PCEA amount (ml)	0.008	0.003	5.569	0.018	1.008	1.001 ~ 1.015

**Fig. 3:**
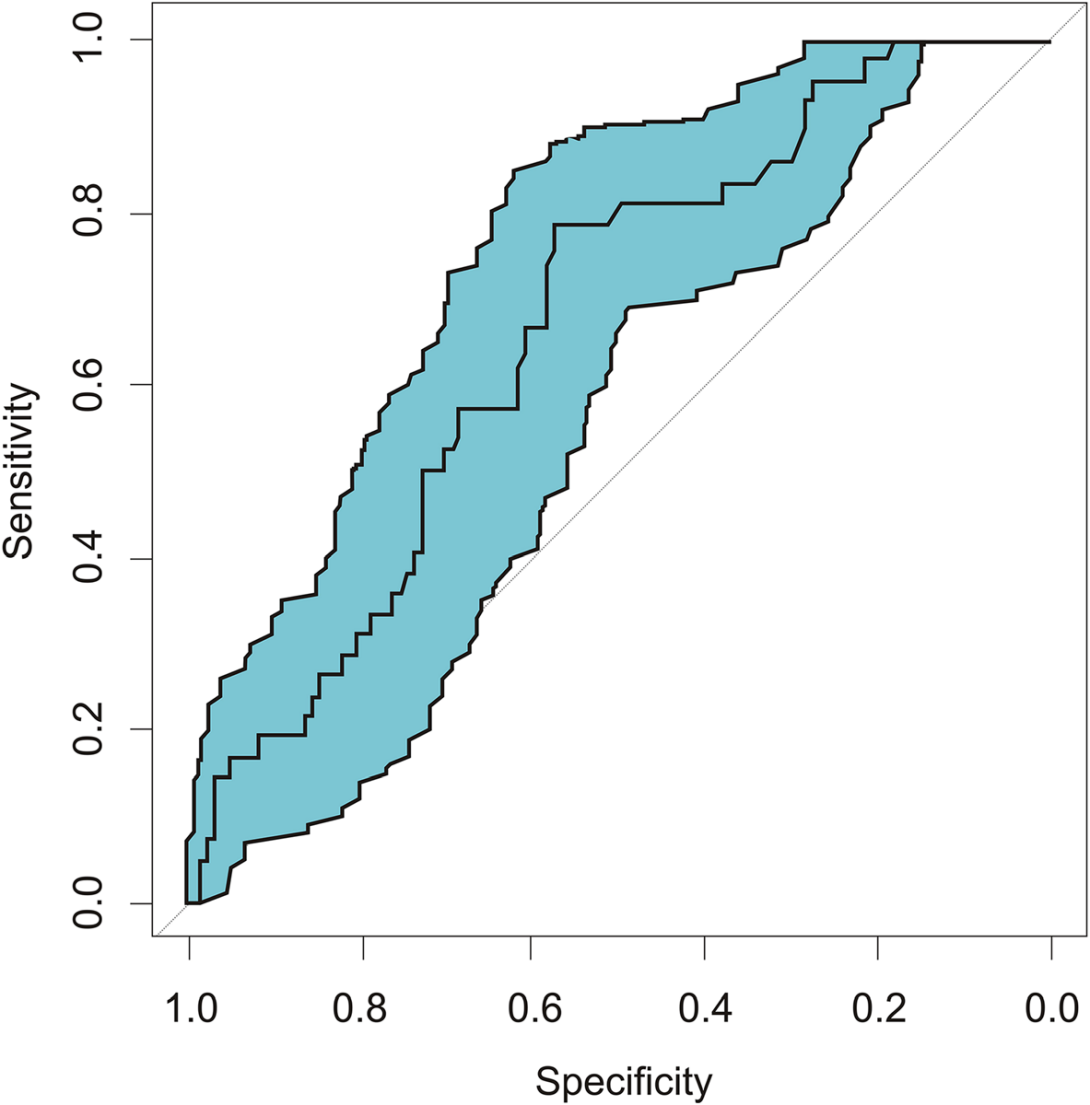
Receiver operating characteristic (ROC) curve for diagnosing fever based on analgesic time. Area under the curve = 0.666, Standard error = 0.045, 95% CI = 0.577–0.755, *P* = 0.001

ROC curve analysis showed that the area under the curve value for diagnosing fever based on the duration of analgesia was 0.666 (0.577 ~ 0.755) (Fig. 3), which was significant (*p < 0.05*). The diagnostic cutoff value for fever was 383 min, with a sensitivity of 78.6% (95% confidence interval [CI]: 65.6–91.5%), specificity of 57.3% (95% CI: 48.2–66.4%), and odds ratio of 4.913 (95% CI: 2.157–11.190) (Fig. 3).

## Discussion

In this study, continuous real-time body temperature monitoring was performed to explore the relationships between the duration and dosage of patient-controlled epidural labor analgesia and intrapartum maternal fever.

The wireless temperature sensor used in this study is an innovative continuous temperature monitoring device that uses a high-precision thermistor probe attached to the axillary root, a model algorithm based on a large number of accumulated experimental data, and computer programs to predict and further, to correct the body temperature [9]. The parameter values recorded by the sensor were assessed from raw measurement data using a noise reduction algorithm (filtered and anti-interfered). The innovative model algorithm was based on a large sample of data collected in the operating room environment such as the room temperature and the human bladder, esophageal, and axillary temperatures, as well as experimental data from different parts of the human body. The sensor accurately reflected the human esophageal temperature. Therefore, the wireless temperature sensor can be considered a convenient and accurate body temperature measurement tool.

There was no significant difference in the general data between the Fever and No-fever groups. Intrapartum maternal fever was time- and dose-dependently correlated with PCEA; parturients with a greater demand for analgesia were more likely to have a fever. Logistic regression analysis showed that the PCEA duration and total PCEA doses were correlated with the incidence of fever, with extremely small effect sizes. The mean temperature-time curves showed a steeper increase of temperature in the Fever group as the duration of analgesia increased. In some cases, when the analgesia duration was greater than 6 h, the maternal temperature was higher than 38 °C. The ROC curve confirmed that the duration of PCEA predicted maternal fever, with epidural analgesia lasting over 6.3 h significantly increasing the risk of fever. Therefore, anesthesiologists and obstetricians should routinely monitor the body temperature of parturients undergoing PCEA, especially when PCEA lasts over 6.3 h and the analgesia demand is high. Early measures should be taken to prevent maternal fever, such as reducing the room temperature, removing clothing, applying fluid hydration, and using oxytocin to accelerate labor.

Other studies found that PCEA was associated with maternal fever, which is an important risk factor for adverse neonatal outcomes, including postpartum hemorrhage and labor dystocia, and is also associated with a higher rate of cesarean section [10]. The effects of fever on newborns include neurodepressive symptoms, such as decreased Apgar scores, a higher likelihood of ventilation and oxygen inhalation, and an increased rate of cardiopulmonary resuscitation [3]. Previously, we observed that women receiving epidural analgesia who developed a fever had a prolonged first stage of labor and increased rates of cesarean section, assisted vaginal delivery, intrapartum and postpartum hemorrhage, and turbid amniotic fluid compared with women without fever, but there were no effects from epidural analgesia on neonate Apgar scores [11]. We believe that it is important to monitor changes in maternal temperature dynamically during labor, and to intervene to ensure the safety of mothers and infants.

Therefore, we continuously monitor the maternal core body temperature and plot a temperature-time curve at our institute. More importantly, we use an information management system during obstetrical delivery to monitor the data from the analgesia pump. This system also enables us to monitor the duration and dosage of PCEA analgesia, and to analyze the correlation between analgesia and body temperature.

Our study confirmed that there were time- and dose-dependent correlations between PCEA and intrapartum maternal fever. However, the influence was weak and predictable. Women with a PCEA duration exceeding 6.3 h were more likely to have a fever. PCEA is an independent risk factor for intrapartum fever, as confirmed by many studies [1, 3, 7, 12]. However, the mechanism by which PCEA causes intrapartum fever is still unclear. Wang et al. randomly divided parturients into an early PCEA group and an IV pethidine with delayed PCEA group [5]. The average analgesic time (12.6 h) in the early PCEA group was significantly longer than that in the delayed group (4.8 h), and the average oral temperatures in the two groups were similar at 37.4 ± 0.4 °C and 37.2 ± 0.3 °C, respectively. In another study, Wang et al. randomly divided parturients into immediate and delayed PCEA groups (average delay, 129 min) after spinal analgesia, and found no significant difference in the maternal fever rate between the groups [6]. Therefore, some researchers speculate that the “trigger-effect” of local anesthetics leads to intrapartum fever via non-infectious inflammation [13].

Others believe that the exposure time and dosage of local anesthetics may also promote maternal fever. Riley et al. found that women with ERMF had higher levels of proinflammatory cytokines (IL-6 and IL-8) even before receiving epidural analgesia, and the subsequent prolonged use of epidural bupivacaine increased the baseline cytokine levels gradually [14]. Zhou et al. found that the IL-6 and tumor necrosis factor (TNF)-α levels of their 0.075% ropivacaine group were significantly lower than those of their 0.1% ropivacaine group [15]. The use of 0.075% ropivacaine caused less inflammation. Lower anesthetic concentrations contributed to lower rates of intrapartum fever. However, they did not report the anesthesia dosage used in their experiment.

In the current clinical study, the duration and total amount of PCEA were both associated with epidural analgesia fever during labor. We speculate that with increasing ropivacaine exposure, the risk of ERMF caused by aseptic inflammation slightly increases. Analysis of the ROC curve showed that an analgesia time longer than 6.3 h significantly increased the risk of fever. Yin et al. reported that the cutoff point for an increased risk of fever was 6 h after analgesia [12]. However, they monitored body temperature hourly, not continuously, and thus could not precisely detect the cutoff point.

Our study had some limitations. First is the study design. This is an observational study rather than a randomized trial, and thus a potential bias may exist. Second, there were no data on the levels of inflammatory factors during delivery, and the time- and dosage-dependent effects of ropivacaine on body temperature were obtained from clinical observations. In addition, the sample size was relatively small. In future research, we will increase the sample size and determine the levels of inflammatory factors.

## Conclusions

This study was conducted with continuous real-time monitoring of maternal core body temperature and the recording of analgesic data during labor, and investigated the associations of PCEA duration and drug dosage with intrapartum maternal fever. The duration of PCEA and total PCEA dose in parturients with fever during delivery were significantly higher than the corresponding values in those without fever. There were weak time- and dose-dependent correlations between PCEA and maternal fever during delivery. Analgesic drugs can cause ERMF and an analgesic time over 6.3 h increases the risk of maternal intrapartum fever to some extent.

## Data Availability

The datasets generated and analyzed during the present study are available from the corresponding author on reasonable request.

## Abbreviations

BMI: Body mass index
ERMF: Epidural-related maternal fever
PCEA: Patient-controlled epidural analgesia
ROC: Receiver operating characteristic
SD: Standard deviation
TNF: Tumor necrosis factor
